# Inhibition of Advanced Glycation End Products (AGEs) Accumulation by Pyridoxamine Modulates Glomerular and Mesangial Cell Estrogen Receptor α Expression in Aged Female Mice

**DOI:** 10.1371/journal.pone.0159666

**Published:** 2016-07-18

**Authors:** Simone Pereira-Simon, Gustavo A. Rubio, Xiaomei Xia, Weijing Cai, Rhea Choi, Gary E. Striker, Sharon J. Elliot

**Affiliations:** 1 Department of Surgery, University of Miami Miller School of Medicine, Miami, Florida, United States of America; 2 Division of Experimental Diabetes and Aging, Department of Geriatrics and Palliative Care, Mount Sinai School of Medicine, 1 Gustave Levy Place, New York, New York, United States of America; 3 Department of Medicine, University of Miami Miller School of Medicine, Miami, Florida, United States of America; Temple University, UNITED STATES

## Abstract

Age-related increases in oxidant stress (OS) play a role in regulation of estrogen receptor (ER) expression in the kidneys. In this study, we establish that *in vivo* 17β-estradiol (E_2_) replacement can no longer upregulate glomerular ER expression by 21 months of age in female mice (anestrous). We hypothesized that advanced glycation end product (AGE) accumulation, an important source of oxidant stress, contributes to these glomerular ER expression alterations. We treated 19-month old ovariectomized female mice with pyridoxamine (Pyr), a potent AGE inhibitor, in the presence or absence of E_2_ replacement. Glomerular ERα mRNA expression was upregulated in mice treated with both Pyr and E_2_ replacement and TGFβ mRNA expression decreased compared to controls. Histological sections of kidneys demonstrated decreased type IV collagen deposition in mice receiving Pyr and E_2_ compared to placebo control mice. In addition, anti-AGE defenses Sirtuin1 (SIRT1) and advanced glycation receptor 1 (AGER1) were also upregulated in glomeruli following treatment with Pyr and E_2_. Mesangial cells isolated from all groups of mice demonstrated similar ERα, SIRT1, and AGER1 expression changes to those of whole glomeruli. To demonstrate that AGE accumulation contributes to the observed age-related changes in the glomeruli of aged female mice, we treated mesangial cells from young female mice with AGE-BSA and found similar downregulation of ERα, SIRT1, and AGER1 expression. These results suggest that inhibition of intracellular AGE accumulation with pyridoxamine may protect glomeruli against age-related oxidant stress by preventing an increase of TGFβ production and by regulation of the estrogen receptor.

## Introduction

Normal aging is associated with an increase in oxidant stress in multiple organs including the kidneys [1, 2]. This effect is observed in both sexes, however, young men have higher levels of oxidant stress markers compared with pre-menopausal age-matched women [3, 4]. These parameters of oxidant stress increase in women after menopause [5]. We previously reported that an age-related increase in oxidant stress mediates a decrease in estrogen receptor alpha (ERα) expression and function in the kidneys [6]. However, the consequences of differences in oxidant stress in the kidneys between pre-and post-menopausal women have not been well-studied.

Advanced glycation end products (AGEs) are a well-known cause of chronic renal oxidant stress and inflammation [7]. Their source is thought to be the high-AGE modern diet [4, 7–9]. Circulating levels of AGEs correlate with the AGE content of common foods, especially those of animal origin [10]. Food AGEs are accumulated by routine methods of industrial and/or home food processing, especially dry heat [11–14]. The amount of orally-absorbed AGEs that interact with tissues is estimated to be 2 to 3-fold greater than the amount in the circulation, an amount that far exceeds the kidney’s excretion capacity [15–17]. Chronic ingestion of excess AGEs is associated with a marked down-regulation of important anti-oxidant defense mechanisms. These include Sirtuin 1 (SIRT1), an NAD^+^-dependent histone deacetylase, advanced glycation receptor 1 (AGER1), and other anti-oxidant systems such as nuclear factor erythroid 2-related factor 2 (Nrf2) [10, 18]. Reduction of renal SIRT1 results in multiple downstream effects including inhibition of ER signaling and reduction of mitochondrial biogenesis and function [19]. In addition, SIRT1 plays a role in preventing NF-*k*B (nuclear factor kappa-light-chain-enhancer of activated B cells) activation, which may also regulate ER expression [20, 21].

In this study, we investigated the potential role of AGEs as a mechanism of glomerular ER regulation. First, we determined the time course of age-related loss of 17β-estradiol (E_2_)-stimulated ER expression regulation in the glomerulus of aged female mice. This phenomenon was observed in 21-month old female mice that were ovariectomized at 19 months at the advent of their anestrous period and prolonged exposure to oxidant stress. We therefore selected 21-month old ovariectomized female mice and fed them a regular mouse diet (high in AGEs) with or without pyridoxamine (Pyr), which is a potent anti-AGE that is currently used in patients with kidney disease. Additional mice were administered E_2_ alone or E_2_ in addition to pyridoxamine. We found that *in vivo* treatment with Pyr and E_2_ increased glomerular ERα expression, while administration of E_2_ alone did not. The combination of Pyr and E_2_ also lowered the glomerular mRNA expression of transforming growth factor beta (TGFβ), a profibrotic cytokine. Moreover, this combination treatment prevented type IV collagen accumulation, which is associated with age-related glomerulosclerosis [22, 23]. SIRT1 and AGER1, important anti-AGE defenses, were upregulated in the Pyr and E_2_ group. Finally, we demonstrate a decrease in ERα and SIRT1 expression in response to AGEs *in vitro* using mesangial cells isolated from young female kidneys, suggesting that AGE accumulation is involved in oxidant stress-related changes in the aged kidney.

## Materials and Methods

### Mice

Female C57Bl/6 mice were obtained from the National Institute of Aging, National Institutes of Health (Bethesda, MD). Mice were ovariectomized at either 12 or 19 months of age using the previously described procedure that has been approved by the Institutional Animal Care and Use Committee at the University of Miami Miller School of Medicine (protocol 12–043) [24]. The mice were divided into 2 groups and received either placebo or 17β-estradiol (E_2_) 90-day release pellets (Innovative Research of America, Sarasota FL) as previously described [25]. The 19-month group was further divided and were provided water with or without pyridoxamine (200 mg/kg per day in 10 ml H_2_O; Biostratum). Mice were euthanized by intraperitoneal injection of ketamine and xylazine as approved by protocol.

#### Mouse Sacrifice

Mice were housed under pathogen-free conditions with food and water ad libitum. Mice were sacrificed 2 months after treatment (at 14 or 21 months of age). Left kidneys were perfused with a buffered solution containing collagenase and RNAse inhibitors for micro dissection of glomeruli, as previously described [25]. Right kidneys were perfused *in situ* with 6 ml of phosphate-buffered saline and 3 ml of 4% paraformaldehyde, post-fixed in 4% paraformaldehyde solution for at least 12 hours and embedded in methacrylate. 4 μm thick sections were stained with periodic acid–Schiff stain. Other kidney fragments were immediately frozen in OCT [26]. Glomeruli were microdissected to isolate mesangial cells from each group.

### Measurements of Urinary Albumin and Creatinine

Spot urine samples were collected at the same hour on a weekly basis and at time of sacrifice. Urine albumin was measured by ELISA following manufacturer’s instructions (Bethyl, Houston, TX) and was corrected for the concentration of urine creatinine. This was expressed as the urinary albumin/creatinine excretion ratio (UAE).

### Kidney tissue histological analysis of type collagen IV

Deparaffinized kidney sections (4 μm) were blocked for endogenous peroxidases. Sections were stained with either rabbit anti-mouse (Biodesign, Saco, ME) or rabbit anti-mouse collage IV. After 1 h, the slides were washed and incubated for 30 min at room temperature with biotinylated-labeled goat anti-rabbit, followed by Vectastain ABC reagent (Vector Labs, Burlingame, CA) and 3,3'-diamino-benzidine chromogen solution (Sigma, St. Louis, MO). The sections were examined and graded on a scale of 0 to 4, as previously described [26], by a renal pathologist (GS) who was blinded to the treatment group.

### Real time PCR

Amplification and measurement of target RNA was performed on the Step 1 Real Time-PCR System, as previously described [25]. The mRNA sequence was obtained from the National Center for Biotechnology Information (Bethesda, MD) to acquire the copy number for each ER subtype, as previously described [27]. The number of occurrences of each of the four nucleobases was counted and multiplied by its respective molecular weight. These four numbers were then summed together to obtain the mass of 1 mol of each subtype of the ER. The mass of the purified plasmid of each subtype and the unknown samples was calculated by the A260 method on a Molecular Devices SpectraMax PLUS (Ramsey, MI, USA) [27]. TGFβ, SIRT1 and AGER1 primers were purchased from Life Technologies (Carlsbad, CA). Specific primer sequences used were as previously described for ER [28], TGF-β [23], SIRT1 and AGER1 [8].

### Isolation of Mesangial Cells

Mesangial cells were isolated from each group of mice treated with and without Pyr in the presence and absence of E_2_ pellets, as previously described [29]. Mesangial cells previously isolated from young female C57/B6 mice (3 months old) were treated with increasing concentrations of AGE-BSA (50–200 μg/ml) to determine effective dose for downregulating ERα protein expression [30]. Once the effective dose was established at 100 μg/ml of AGE-BSA, cells were treated with AGE-BSA for 24 hours. This treatment time frame was determined by exposing cells to increasing time intervals (2–48 hours) of AGE-BSA and determining its effect on ERα protein expression.

### Western Blot Analysis

For protein analyses, cell lysates were extracted and protein quantity assessed using the Pierce BCA protein assay kit (Rockford, IL). Equal amounts of protein were applied to precast SDS polyacrylamide gels (Life Technologies, Grand Island, NY) and analyzed as previously described for ERα, AGER1, SIRT1, and β-actin [31]. In some experiments, cells were treated overnight with AGE-BSA (100 μg/ml for 18 hours). Western blots were also exposed to β-actin (Sigma Chemical, St. Louis MO.) to control for protein loading. Human recombinant ERα was used as a control (PanVera, Madison, WI). Immunoreactive bands were determined by exposing nitrocellulose blots to a chemiluminescent solution (Denville Scientific Inc., Metuchen, NJ) followed by exposure to Amersham Hyperfilm ECL (GE Healthcare Limited, Buchinghamshire, UK). Relative amounts of protein were determined by densitometry using ImageJ software version 1.48 (National Institutes of Health, Bethesda, MD).

### Statistical analysis

All values are expressed as mean ± standard error of the mean (SEM). Significance of overall differences within experimental groups was determined by analysis of variance (ANOVA) in combination of Tukey’s multiple comparison test. Student’s t-test was used to determine differences between groups, using Welch’s correction as appropriate. P values < 0.05 were considered significant.

## Results

### Glomerular ERα mRNA upregulation by 17β-estradiol replacement is lost by anestrous period (21 months of age)

**Fig 1:**
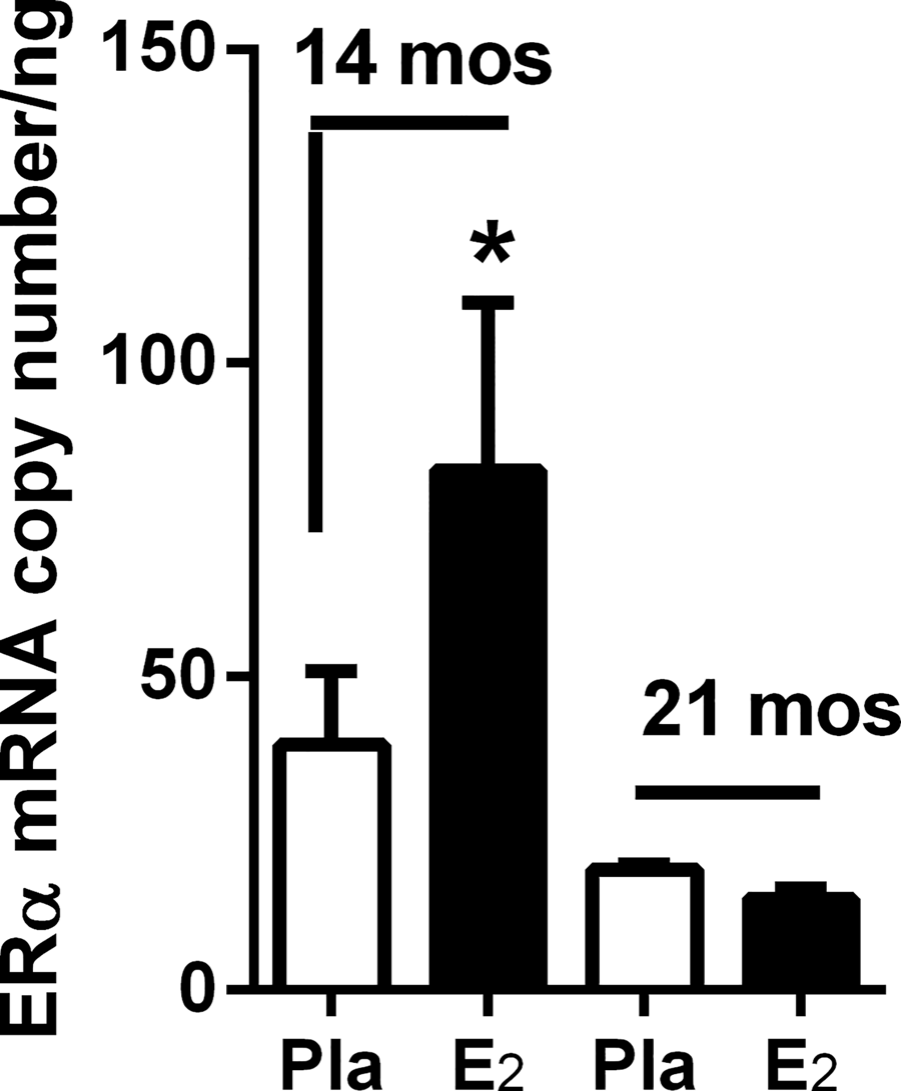
Glomerular ERα mRNA upregulation by 17β-estradiol replacement is lost by anestrous period (21 months of age). ERα mRNA copy number was determined by real-time-PCR of glomeruli isolated from female C57BL6 mice ovariectomized at 12-, and 19-months of age (n = 5/group). E_2_ was administered within 2 weeks of ovariectomy and mice sacrificed at 14 and 21 months. Data are graphed as mean ± SEM. *p < 0.05, compared with placebo.

To determine the time course of age-related loss of 17β-estradiol (E_2_)-stimulated glomerular ER expression regulation, we replaced E_2_ for 2 months in 12-month old (pre-anestrous) and 19-month old (anestrous) female mice. All mice were ovariectomized two weeks prior to E_2_ administration to ensure equivalent replacement. E_2_ replacement was only effective in up regulating glomerular ERα mRNA expression in 12-month old mice prior to entering the anestrous period (at approximately 18 months of age), and thus correlating with a shorter exposure to endogenous oxidant stress (Fig 1). By 21 months of age (anestrous) E_2_ replacement failed to upregulate ERα mRNA expression (Fig 1).

### Effect of pyridoxamine and 17β-estradiol replacement on body, kidney, uterine weight and albumin/creatinine ratio

Treatment of mice did not alter body weight, however, kidney weight increased in Pyr+E_2_ treatment compared to placebo and Pyr alone (*p<0.05). Uterine weight as a marker of estrogen replacement was increased in all mice receiving E_2_ regardless of whether they were also receiving Pyr (Table 1). Urinary albumin excretion did not change between groups (Table 1).

**Table 1:** Effect of pyridoxamine and 17β-estradiol replacement on body, kidney, uterine weight and albumin/creatinine ratio.

	Pla (n = 10)	E_2_ (n = 12)	Pyr (n = 10)	Pyr+E_2_ (n = 6)
**Body weight (g)**	31 ±1.3	30±0.9	31±1.5	30±1.4
**Kidney weight (g)**	0.28±0.01	0.29±0.01	0.27±0.009	0.34±0.2 ^**a**^
**Uterine weight (g)**	0.02±0.00^**b**^	0.14±0.02^**c**^	0.02±0.001^**d**^	0.15±0.02
**Albumin/Creatinine ratio**	0.43±0.32	0.26±0.06	0.34±0.06	0.25±0.05

a *p<0.05 compared to placebo (pla) and pyridoxamine (Pyr)

b***p<0.005 compared to 17β-estradiol (E_2_) and Pyr+E_2_

c***p<0.005 compared to Pyr

d ***p<0.005 compared to Pyr+E_2_.

### Inhibition of AGE accumulation with pyridoxamine and 17β-estradiol increases glomerular ERα mRNA expression and reduces TGFβ mRNA expression

**Fig 2:**
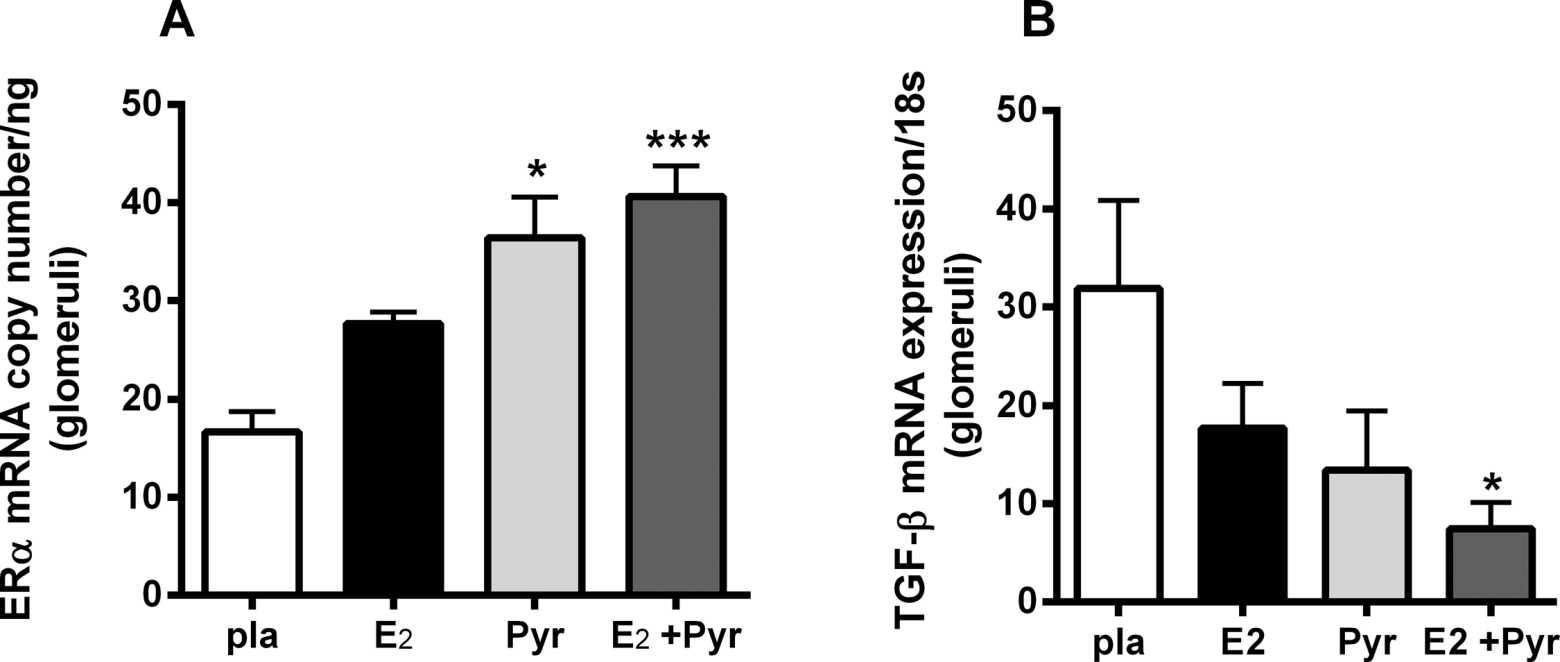
17β-estradiol (E_2_) combined with anti-AGE treatment pyridoxamine (Pyr) increases ER and decreases TGF mRNA in glomeruli of aged ovariectomized female mice. 21 month-old ovariectomized female C57/B6 mice were treated with either placebo (pla), 17-estradiol (E_2_), pyridoxamine (Pyr) or E_2_+ Pyr for 2 months. Real time-PCR for ER and TGF were performed as described in methods. Data are graphed as mean ± SEM.*p <0.05 compared to pla, ***p<0.005 compared to pla, # p<0.05 compared to pla. n = 5–7 mice/group.

Our previous study showed an oxidant stress-related glomerular ERα downregulation associated with aging [6]. In this study, *in vivo* inhibition of AGEs, a source of oxidant stress, with Pyr and E_2_ administration increased ERα mRNA expression (Fig 2A) in 21 month-old ovariectomized female mice. TGFβ, a profibrotic cytokine, was decreased in an inverse manner to ERα mRNA expression in the group receiving Pyr and E_2_ (Fig 2B). There was no significant difference in ERα or TGFβ mRNA expression between placebo group and mice receiving either Pyr or E_2_ alone.

### Type IV collagen deposition decreases with pyridoxamine and 17β-estradiol treatment in aged estrogen-deficient female mice

**Fig 3:**
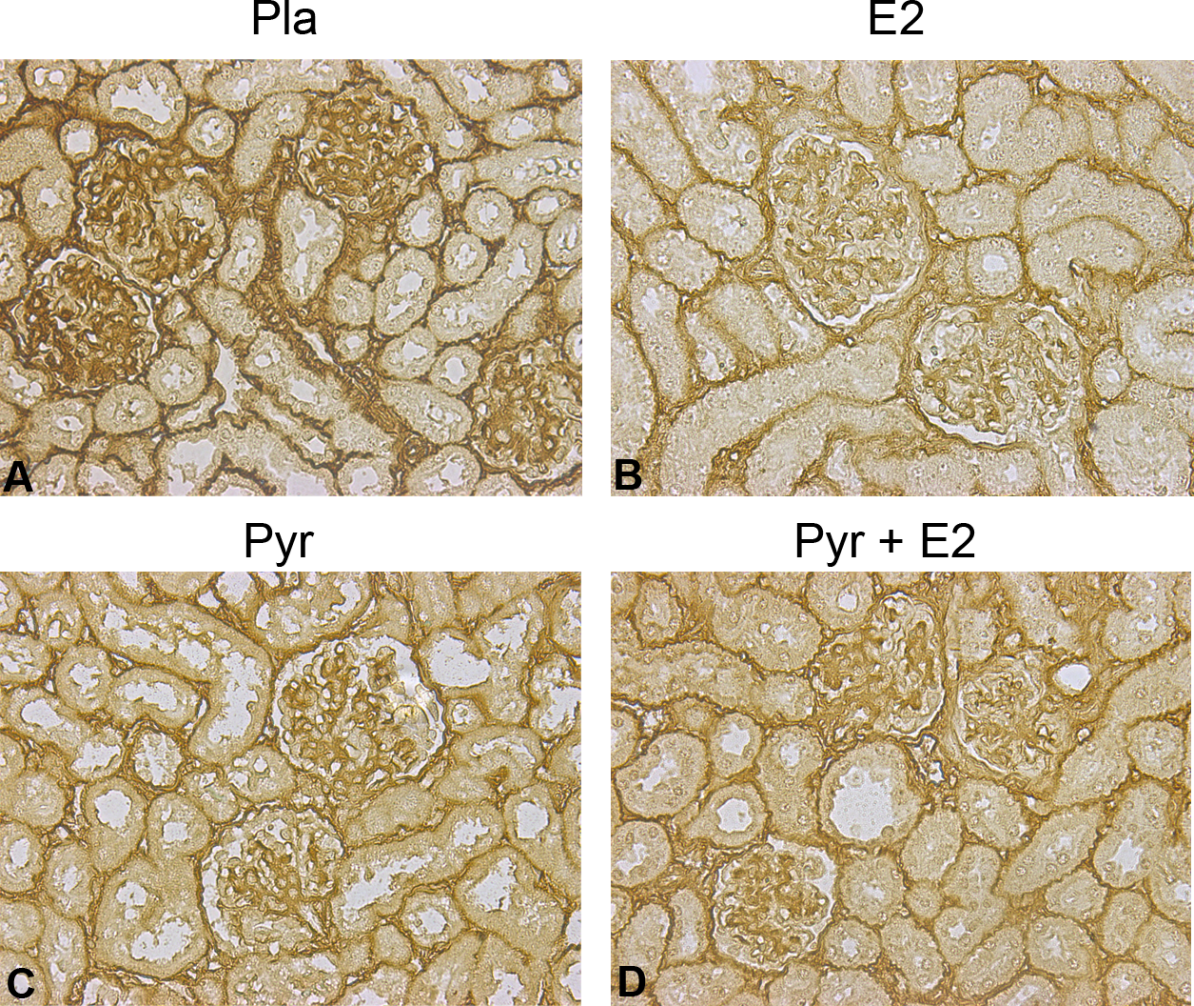
Age-related glomerular collagen deposition is reduced after pyridoxamine (Pyr) and 17β-estradiol treatment. Collagen type IV deposition is increased in ovariectomized old female C57/B6 mice (placebo control, panel A). 17β-estradiol (E_2_) treatment (panels B and D) prevented collagen accumulation, particularly in combination with pyridoxamine (Pyr) treatment (panel D). All mice were rendered estrogen deficient by ovariectomy at 19 months of age. Images are representative of staining that was performed on at least five mice per group. Magnification, 40x.

Type IV collagen, one of the hallmarks of glomerulosclerosis, increased in placebo-treated glomeruli of ovariectomized aged female mice as expected (3+ staining; Fig 3A). Treatment with the antioxidant pyridoxamine decreased the accumulation of type IV collagen in glomeruli and tubules (1 and 2+ staining; Fig 3C). E_2_ replacement, with or without pyridoxamine, also prevented accumulation of type IV collagen in estrogen-deficient (ovariectomized) aged female mice (1 and 2+ staining; Fig 3B–3D).

### Prevention of AGE accumulation with pyridoxamine in the presence of E2 replacement increases glomerular SIRT1 and AGER1 mRNA

**Fig 4:**
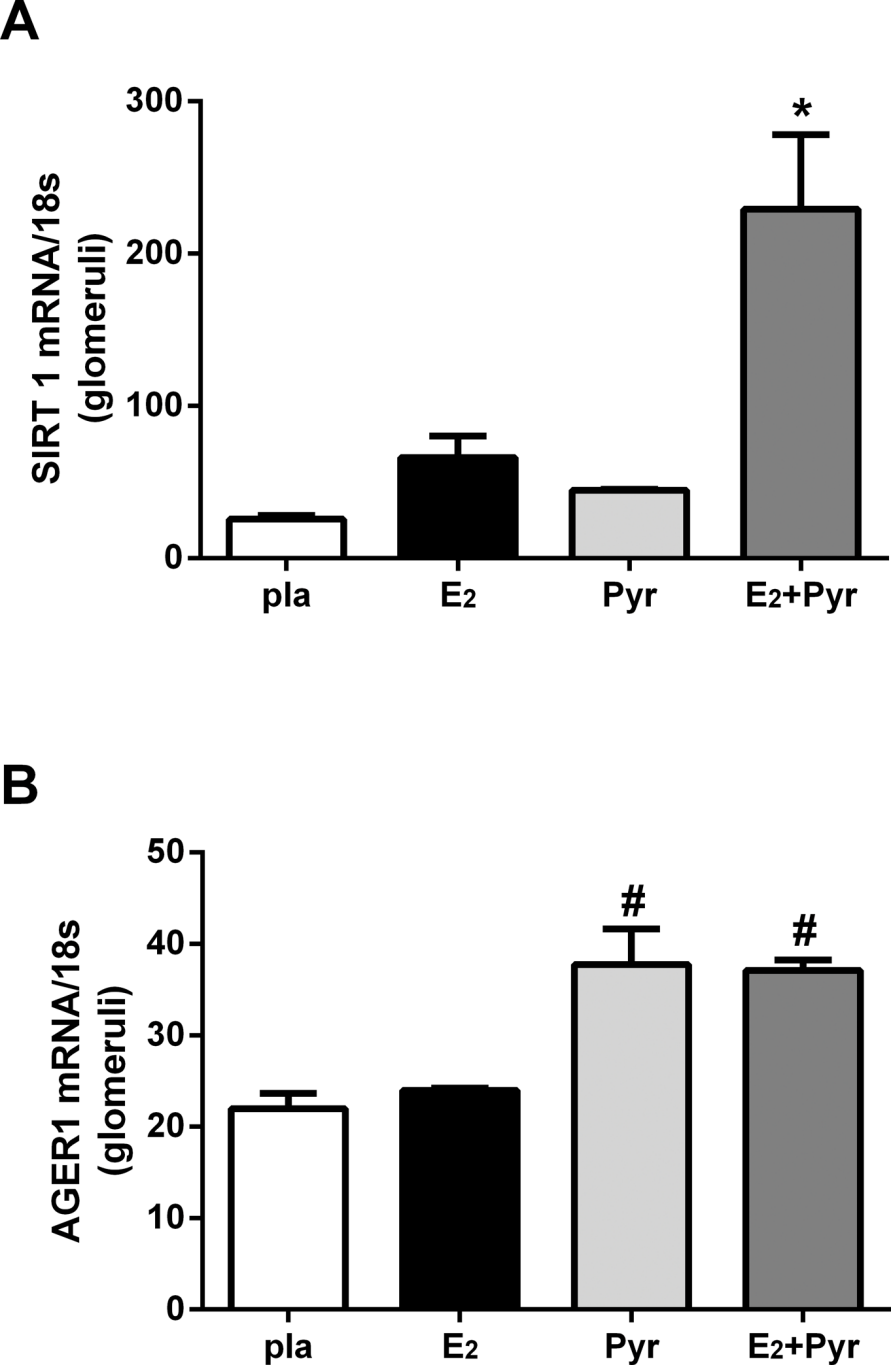
Glomerular AGER1 and SIRT-1 mRNA are upregulated by reduction of AGEs *in vivo*. Glomeruli were isolated from 4 groups of mice; placebo (pla), 17β-estradiol (E_2_), pyridoxamine (Pyr) or E_2_+ Pyr. SIRT1, AGER1 and 18s were measured by RT-PCR as described in Methods. Data are graphed as mean ± SEM of ratio of SIRT1/18s (*p<0.05 compared to all groups) or AGER1/18s (#p<05 compared to placebo and E_2_ treatments). n = 5/group.

AGE accumulation down-regulates anti-oxidant stress defenses such as SIRT1 and AGER1 [8, 18]. Therefore, we measured glomerular SIRT1 and AGER1 mRNA expression in our 4 groups of ovariectomized 21-month old female mice. Glomerular expression of SIRT1 mRNA was increased in mice treated with pyridoxamine and E_2_ replacement compared to all other groups (Fig 4A, *p < 0.05). Similarly, AGER1 mRNA expression was increased in the glomeruli of mice receiving pyridoxamine and E_2_ replacement compared to placebo or E_2_ alone groups (Fig 4B, #p < 0.05). AGER1 expression also increased in mice treated with pyridoxamine alone (Pyr) versus placebo or E_2_ alone (Fig 4B, #p < 0.05).

### Mesangial cells isolated from aged female mice treated with Pyr + E2 maintain a phenotypic switch with increased ERα, SIRT1 and AGER1 mRNA expression

**Fig 5:**
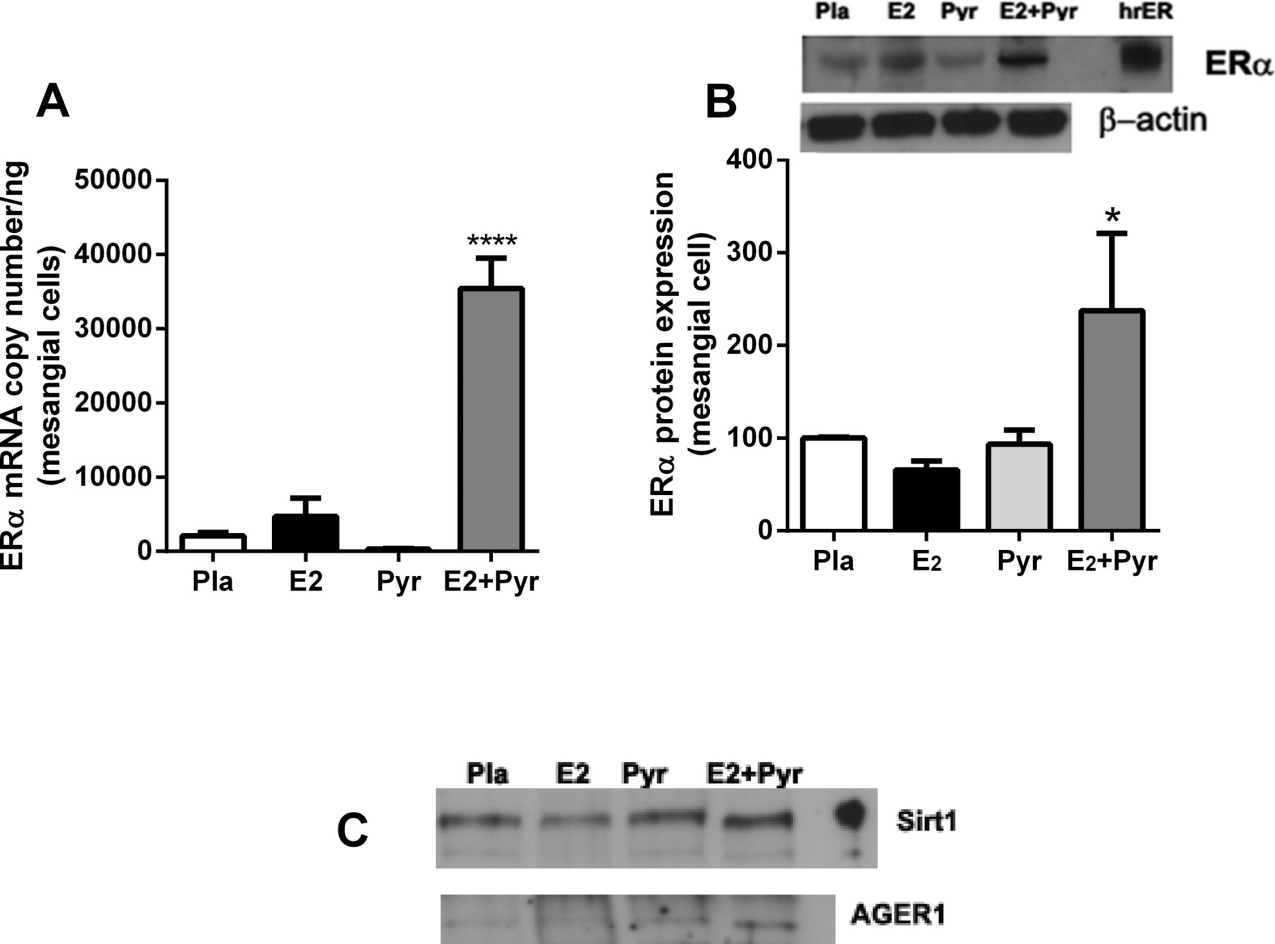
Mesangial cells isolated from aged female mice treated with pyridoxamine and 17β-estradiol maintain a phenotypic switch with increased ERα, SIRT1 and AGER1 mRNA expression. **A)** Mesangial cell ERα mRNA copy number was measured on isolates by RT-PCR as described in Methods. ****p<0.0001 compared to all groups. **B + C)** Mesangial cell protein expression of ERα (Fig 5B), SIRT-1 and AGER1 (Fig 5C) was measured by western blot from each group of treated mice. Shown is a representative Western blot and β-actin loading control. For ERα data are graphed as mean ± SEM % of placebo control. *p<0.05 compared to all groups. n = 2 isolates/group.

At the time of sacrifice, glomeruli were isolated and cells propagated from the four groups of mice described above. ERα mRNA copy number and protein expression was increased only in mesangial cells isolated from mice that were treated with both Pyr and E_2_ (Fig 5A and 5B). Similarly, we found an increase in SIRT1 and AGER1 protein expression in cells derived from mice treated with Pyr + E_2_ (Fig 5C).

### AGEs reduce glomerular ERα protein expression *in vitro*

**Fig 6:**
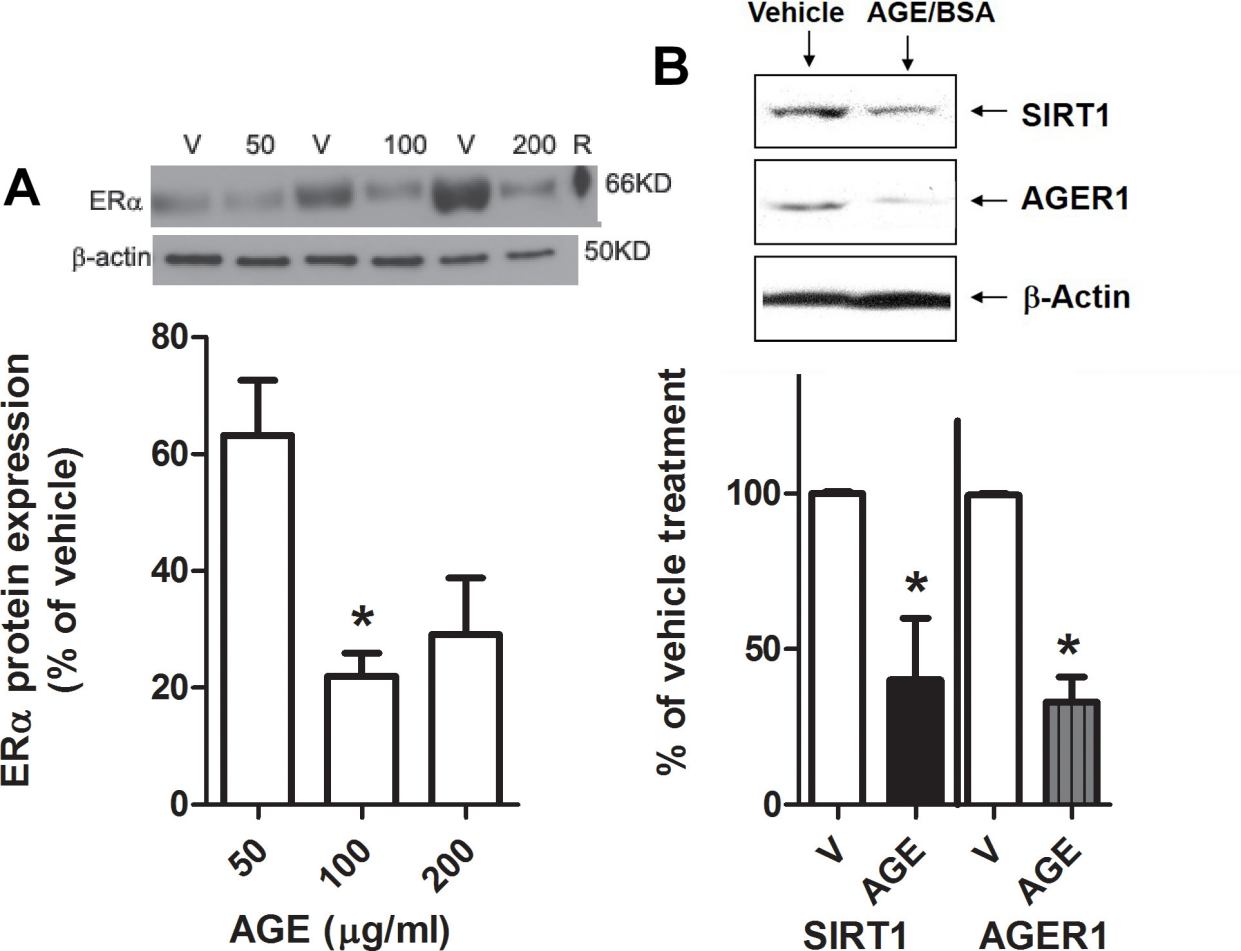
**Advanced glycation end products (AGEs) suppress ER protein levels in mesangial cells: A)** Western analysis of ERα protein expression in mesangial cells isolated from young female mice treated with increasing doses of AGE-BSA. Density data show % of control BSA (or vehicle, V) and graphed as mean ± SEM, *p<0.05 compared to 50 μg/ml. B) Western blot of SIRT1, AGER1 and -actin in mesangial cells from young female mice treated with 100 μg/ml of AGE-BSA for 24 hours. Density data graphed as mean ± SEM % of BSA (vehicle or V). *p<0.05 compared to vehicle, n = 6.

To further confirm that AGEs reduce glomerular ERα expression, mesangial cells isolated from young female mice were treated with AGEs *in vitro*. ERα protein expression was decreased after treatment with AGE-BSA (Fig 6A). There was also a decrease in SIRT1 and AGER1 protein expression in these cells (Fig 6B).

## Discussion

We have previously shown that E_2_ upregulates glomerular ERα mRNA and protein expression in young mice [28], but during aging there is a steady decline in both [6]. In the present study, we demonstrate that timing of estrogen replacement in relation to reproductive age is critical for regulation of glomerular ER expression. E_2_ replacement at 14 months (before anestrus) was effective in upregulating ERα. This effect, however, was lost by 21 months of age coinciding with the anestrus period and prolonged exposure to oxidant stress. These data derived in experimental animals may provide insight into the findings of the Women’s Health Initiative (WHI) and Heart and Estrogen/Progestin Replacement Study (HERS). In those trials, women that received estrogen replacement up to 10 years after menopause exhibited some adverse clinical outcomes. The KEEPs trial, on the other hand, studied women not more than three years post menopause and suggested that this window of time for initiation of hormone replacement may lead to a beneficial effect for disease prevention [32]. Our previous data showed that increased oxidant stress is associated with reduced ERα expression in the kidney of aging mice [6]. Therefore, it is possible that administration of estrogen during this time of increased age-related oxidant stress leading to decrease in ER expression and action may exacerbate downstream deleterious events.

Based on our previous findings, we designed the current study to further investigate the role of oxidant stress and regulation of glomerular ERα expression *in vivo*. We examined the effect on glomerular ERα expression of pyrydoxamine, a derivative of vitamin B_6_ that prevents intracellular accumulation of AGEs and scavenges reactive oxygen species [33]. Pyridoxamine treatment coupled with E_2_ replacement increased glomerular ERα expression, while E_2_ replacement alone did not. Furthermore, ERα expression in mesangial cells isolated from *in vivo* treated mice followed a similar expression pattern as in the glomeruli. This was expected, as we have previously reported that a phenotypic switch in glomerular ERα expression occurring *in vivo* is maintained *in vitro* [25, 34].

Aged female mice (24 months of age and older) have increased urinary albumin excretion and collagen types I and IV deposition leading to glomerulosclerosis [23]. This increase in glomerulosclerosis markers associated with age can be observed in experimental models and humans [23] [35, 36]. Although baseline urinary albumin excretion was higher in our aged female mice compared to young female mice (data not shown), this was not affected by treatment with pyridoxamine and/or E_2_ in aged ovariectomized female mice. It is possible that prolonged treatment period and sacrifice at an older age may have revealed an effect. In contrast, all treatment combinations prevented glomerular type IV collagen deposition in aged females. Of note, despite the effectiveness of oral pyridoxamine in preserving kidney function in type 1 and 2 diabetic rat and mouse models [37–39], recent clinical trials in patients with type 1 and type 2 diabetes produced mixed results [40, 41]. Williams et al. [40] showed a reduction of baseline serum creatinine without a change in urine albumin excretion. A larger study failed to show any change in renal function after 1 year, although the authors suggested that patients with less severe renal damage may respond to the drug [41].

In the present study, *in vivo* pyridoxamine treatment along with E_2_ replacement decreased TGFβ mRNA expression in kidneys of aged ovariectomized female mice. Accumulation of gene expression of growth factors and cytokines such as TGFβ and vascular endothelial growth factor (VEGF) are associated with the formation of AGEs [42]. We and others have shown that kidney disease in mice and humans is often associated with increased TGFβ expression [26, 43–45]. In fact, TGFβ signaling can be initiated by reactive oxygen species, which could ultimately increase extracellular matrix protein (ECM) accumulation through direct upregulation of collagen synthesis and/or decreased matrix metalloproteinase activity. In addition, TGF-β1 contributes to glomerulosclerosis by stimulating podocyte apoptosis [44, 46]. Finally, TGF-β receptor 2 is increased in isolated mesangial cells and in glomeruli of diabetic mice, suggesting an increased sensitivity due to the effects of endogenous TGF-β1 [47, 48]. Interestingly, there was an inverse relationship in our study between the NAD+-dependent deacetylase SIRT1 and TGFβ expression. Negative cross-talk between TGFβ signaling and SIRT has been previously demonstrated in the kidney, liver, and lung [49–51]. SIRTs have been shown to downregulate TGFβ either by degradation or inhibition of transcriptional activity and further studies are ongoing in our laboratory to understand these findings.

SIRT1 and ERα expression were positively correlated in both glomeruli and mesangial cells. We postulate that SIRT may have a direct effect on ER regulation. Estrogen receptors are dynamically modulated by post-translational modification, i.e. phosphorylation, methylation, acetylation, ubiquitination, or sumoylation [52]. For instance, hyperactivation of ERK/MAPK (Extracellular-signal-regulated kinases/Mitogen-activated protein kinases) causes functional repression of ER transcription through NF*k*B activation [53, 54], which we have shown to be increased in 28-month old female mice [22]. In contrast, SIRT1 prevents undue activation of NF*k*B [55, 56]. AGEs promote NF*k*B activation [57] but suppress SIRT1 and its deacetylase activity on NF*k*B-p65 [58]. This could influence ER transcription, given that decreased SIRT1 expression can disrupt the basal transcription factor complex of ERα promoter in some cells [20]. These studies are currently under investigation.

The concentration of AGEs and their cross-linked products increases with aging and leads to higher basal levels of oxidant stress [10, 59]. Importantly, levels of AGEs are elevated in post-menopausal women compared to healthy young women. This increase is more pronounced in diabetic post-menopausal women [3–5]. These data correlate with the higher female to male ratio in patients with diabetic end-stage renal disease, which increases sharply in the postmenopausal age groups [60]. To confirm our *in vivo* data suggesting an important role for AGEs in regulation of glomerular ERα expression in aged females, we examined the direct effects of AGEs *in vitro*. Mesangial cells isolated from young (estrogen replete) female mice were treated with increasing concentrations of AGEs. We observed a dose- and time-dependent reduction in ERα expression in response to AGEs. Similarly, levels of the major cellular anti-AGE/oxidant stress defenses, anti-AGE receptor AGER1 and SIRT1 protein expression were decreased in response to AGE. This correlates with the inverse relationship between SIRT1/AGER1 and AGEs both in the current study and other experimental and human studies [2, 8, 61].

In summary, the ability of pyridoxamine to reverse a fibrotic marker of glomerulosclerosis (TGFβ) and ERα expression in aged female mice (21 months old) suggests that oxidant stress-related damage in the aging kidney is reversible. Furthermore, it is possible that reduced anti-oxidant defenses, such as SIRT1 and AGER1, in postmenopausal women could impair glomerular E_2_/ER activity.

## References

[1] Lee HC, Wei YH. Oxidative stress, mitochondrial DNA mutation, and apoptosis in aging. ExpBiolMed(Maywood). 2007;232(5):592–606. 17463155

[2] Cai W, He JC, Zhu L, Chen X, Wallenstein S, Striker GE, et al Reduced oxidant stress and extended lifespan in mice exposed to a low glycotoxin diet: association with increased AGER1 expression. AmJPathol. 2007;170(6):1893–902. 10.2353/ajpath.2007.061281 PMC1899464 17525257

[3] Loft S, Vistisen K, Ewertz M, Tjonneland A, Overvad K, Poulsen HE. Oxidative DNA damage estimated by 8-hydroxydeoxyguanosine excretion in humans: influence of smoking, gender and body mass index. Carcinogenesis. 1992;13(12):2241–7. 1473230 10.1093/carcin/13.12.2241

[4] Uribarri J, Cai W, Peppa M, Goodman S, Ferrucci L, Striker G, et al Circulating glycotoxins and dietary advanced glycation endproducts: two links to inflammatory response, oxidative stress, and aging. J GerontolA BiolSci MedSci. 2007;62(4):427–33. 10.1093/gerona/62.4.427 PMC2645629 17452738

[5] Helmersson J, Mattsson P, Basu S. Prostaglandin F(2alpha) metabolite and F(2)-isoprostane excretion rates in migraine. ClinSci (Lond). 2002;102(1):39–43. 11749659

[6] Pereira-Simon S, Xia X, Catanuto P, Elliot S. Oxidant Stress and Mitochondrial Signaling Regulate Reversible Changes of ERa Expression and Apoptosis in Aging Mouse Glomeruli and Mesangial Cells. Endocrinology. 2012;153((11)):5491–9. 10.1210/en.2012-1379 23027807 PMC3473210

[7] Koschinsky T, He CJ, Mitsuhashi T, Bucala R, Liu C, Buenting C, et al Orally absorbed reactive glycation products (glycotoxins): an environmental risk factor in diabetic nephropathy. ProcNatlAcadSci USA. 1997;94(12):6474–9. 10.1073/pnas.94.12.6474 PMC21074 9177242

[8] Cai W, Ramdas M, Zhu L, Chen X, Striker GE, Vlassara H. Oral advanced glycation endproducts (AGEs) promote insulin resistance and diabetes by depleting the antioxidant defenses AGE receptor-1 and sirtuin 1. Proc NatlAcadSciUSA. 2012. 10.1073/pnas.1205847109 PMC3465382 22908267

[9] Vlassara H, Cai W, Goodman S, Pyzik R, Yong A, Chen X, et al Protection against loss of innate defenses in adulthood by low advanced glycation end products (AGE) intake: role of the antiinflammatory AGE receptor-1. J Clin Endocrinol Metab. 2009;94(11):4483–91. 10.1210/jc.2009-0089 19820033 PMC2775660

[10] Vlassara H, Striker GE. AGE restriction in diabetes mellitus: a paradigm shift. Nat Rev Endocrinol. 2011;7(9):526–39. 10.1038/nrendo.2011.74 21610689 PMC3708644

[11] Cai W, Gao QD, Zhu L, Peppa M, He C, Vlassara H. Oxidative stress-inducing carbonyl compounds from common foods: novel mediators of cellular dysfunction. Mol Med. 2002;8(7):337–46. 12393931 PMC2040002

[12] Brands CM, Alink GM, van Boekel MA, Jongen WM. Mutagenicity of heated sugar-casein systems: effect of the Maillard reaction. J AgricFood Chem. 2000;48(6):2271–5. 10.1021/jf9907586 10888535

[13] Finot PA. Historical perspective of the Maillard reaction in food science. AnnN YAcadSci. 2005;1043:1–8. 10.1196/annals.1333.001 16037216

[14] Pouillart P, Mauprivez H, it-Ameur L, Cayzeele A, Lecerf JM, Tessier FJ, et al Strategy for the study of the health impact of dietary Maillard products in clinical studies: the example of the ICARE clinical study on healthy adults. AnnN YAcadSci. 2008;1126:173–6. 10.1196/annals.1433.040 18448812

[15] Goldberg T, Cai W, Peppa M, Dardaine V, Baliga BS, Uribarri J, et al Advanced glycoxidation end products in commonly consumed foods. J Am DietAssoc. 2004;104(8):1287–91. 10.1016/j.jada.2004.05.214 15281050

[16] Uribarri J, Tuttle KR. Advanced glycation end products and nephrotoxicity of high-protein diets. ClinJ Am Soc Nephrol. 2006;1(6):1293–9. 17699361 10.2215/CJN.01270406

[17] Kosch M, Levers A, Fobker M, Barenbrock M, Schaefer RM, Rahn K, et al Dialysis filter type determines the acute effect of haemodialysis on endothelial function and oxidative stress. Nephrology Dialysis Transplantation. 2003;18(7):1370–5. 10.1093/ndt/gfg169 12808175

[18] Uribarri J, Cai W, Ramdas M, Goodman S, Pyzik R, Chen X, et al Restriction of advanced glycation end products improves insulin resistance in human type 2 diabetes: potential role of AGER1 and SIRT1. Diabetes Care. 2011;34(7):1610–6. 10.2337/dc11-0091 21709297 PMC3120204

[19] Nogueiras R, Habegger KM, Chaudhary N, Finan B, Banks AS, Dietrich MO, et al Sirtuin 1 and Sirtuin 3: Physiological Modulators of Metabolism. Physiological Reviews. 2012;92(3):1479–514. 10.1152/physrev.00022.2011 22811431 PMC3746174

[20] Yao Y, Li H, Gu Y, Davidson NE, Zhou Q. Inhibition of SIRT1 deacetylase suppresses estrogen receptor signaling. Carcinogenesis. 2010;31(3):382–7. 10.1093/carcin/bgp308 19995796 PMC2832546

[21] Moore RL, Dai Y, Faller DV. Sirtuin 1 (SIRT1) and steroid hormone receptor activity in cancer. Journal of Endocrinology. 2012;213(1):37–48. 10.1530/JOE-11-0217 22159506 PMC3804056

[22] Zheng F, Cheng QL, Plati AR, Ye SQ, Berho M, Banerjee A, et al The glomerulosclerosis of aging in females: contribution of the proinflammatory mesangial cell phenotype to macrophage infiltration. AmJPathol. 2004;165(5):1789–98. 10.1016/S0002-9440(10)63434-7 PMC1618669 15509547

[23] Zheng F, Plati AR, Potier M, Schulman Y, Berho M, Banerjee A, et al Resistance to glomerulosclerosis in B6 mice disappears after menopause. AmJPathol. 2003;162(4):1339–48. 10.1016/S0002-9440(10)63929-6 PMC1851217 12651625

[24] Elliot SJ, Berho M, Korach K, Doublier S, Lupia E, Striker GE, et al Gender-specific effects of endogenous testosterone: Female alpha-estrogen receptor-deficient C57Bl/6J mice develop glomerulosclerosis. Kidney Int. 2007;72(4):464–72. 17495854 10.1038/sj.ki.5002328

[25] Karl M, Berho M, Pignac-Kobinger J, Striker GE, Elliot SJ. Differential effects of continuous and intermittent 17beta-estradiol replacement and tamoxifen therapy on the prevention of glomerulosclerosis: modulation of the mesangial cell phenotype in vivo. AmJPathol. 2006;169(2):351–61. 10.2353/ajpath.2006.051255 PMC1698782 16877338

[26] Elliot SJ, Karl M, Berho M, Xia X, Pereria-Simon S, Espinosa-Heidmann D, et al Smoking induces glomerulosclerosis in aging estrogen-deficient mice through cross-talk between TGF-beta1 and IGF-I signaling pathways. JAmSocNephrol. 2006;17(12):3315–24. 10.1681/ASN.2006070799 17093064

[27] C P., Doublier S, Fornoni A, Lupia E, Berho M, Striker GE, et al 17b-estradiol and Tamoxifen upregulate estrogen receptor â and regulate podocyte signaling pathways in a model of type 2 diabetes. Kidney International. 2009;75:1194–201. 10.1038/ki.2009.69 19279558

[28] Potier M, Elliot SJ, Tack I, Lenz O, Striker GE, Striker LJ, et al Expression and regulation of estrogen receptors in mesangial cells: influence on matrix metalloproteinase-9. JAmSocNephrol. 2001;12(2):241–51. 10.1681/ASN.V122241 11158214

[29] MacKay K, Striker LJ, Elliot S, Pinkert CA, Brinster RL, Striker GE. Glomerular epithelial, mesangial, and endothelial cell lines from transgenic mice. Kidney Int. 1988;33(3):677–84. . 2835539 10.1038/ki.1988.53

[30] Cai W, He JC, Zhu L, Lu C, Vlassara H. Advanced glycation end product (AGE) receptor 1 suppresses cell oxidant stress and activation signaling via EGF receptor. Proc NatlAcadSci USA. 2006;103(37):13801–6. 10.1073/pnas.0600362103 PMC1564251 16954185

[31] Potier M, Karl M, Zheng F, Elliot SJ, Striker GE, Striker LJ. Estrogen-Related Abnormalities in Glomerulosclerosis-Prone Mice:Reduced Mesangial Cell Estrogen Receptor Expression and Prosclerotic Response to Estrogens. Am J Path. 2002;160:1877–85. 12000739 10.1016/S0002-9440(10)61134-0 PMC1850880

[32] Wharton W, Gleason CE, Miller VM, Asthana S. Rationalle and design of the Kronos Early Estrogen Prevention Study (KEEPS) and the KEEPS cognitive and affective sub study (KEEPS Cog). Brain Research. (0). 10.1016/j.brainres.2013.04.011 PMC3731133 23603409

[33] Kang Z, Li H, Li G, Yin D. Reaction of pyridoxamine with malondialdehyde: mechanism of inhibition of formation of advanced lipoxidation end-products. AminoAcids. 2006;30(1):55–61. 10.1007/s00726-005-0209-6 15990947

[34] Elliot SJ, Striker LJ, Hattori M, Yang CW, He CJ, Peten EP, et al Mesangial cells from diabetic NOD mice constitutively secrete increased amounts of insulin-like growth factor-I. Endocrinology. 1993;133(4):1783–8. 7691581 10.1210/endo.133.4.7691581

[35] Eikmans M, Baelde HJ, de Heer E, Bruijn JA. Effect of age and biopsy site on extracellular matrix mRNA and protein levels in human kidney biopsies. Kidney Int. 2001;60(3):974–81. 11532092 10.1046/j.1523-1755.2001.060003974.x

[36] Kalant N, Satomi S, White R, Tel E. Changes in renal glomerular basement membrane with age and nephritis. CanJ Biochem. 1977;55(12):1197–206. 597769 10.1139/o77-179

[37] Alderson NL, Chachich ME, Frizzell N, Canning P, Metz TO, Januszewski AS, et al Effect of antioxidants and ACE inhibition on chemical modification of proteins and progression of nephropathy in the streptozotocin diabetic rat. Diabetologia. 2004;47(8):1385–95. 15309289 10.1007/s00125-004-1474-8

[38] Alderson NL, Chachich ME, Youssef NN, Beattie RJ, Nachtigal M, Thorpe SR, et al The AGE inhibitor pyridoxamine inhibits lipemia and development of renal and vascular disease in Zucker obese rats. Kidney Int. 2003;63(6):2123–33. 12753299 10.1046/j.1523-1755.2003.00027.x

[39] Tanimoto M, Gohda T, Kaneko S, Hagiwara S, Murakoshi M, Aoki T, et al Effect of pyridoxamine (K-163), an inhibitor of advanced glycation end products, on type 2 diabetic nephropathy in KK-A(y)/Ta mice. Metabolism. 2007;56(2):160–7. 17224327 10.1016/j.metabol.2006.08.026

[40] Williams ME, Bolton WK, Khalifah RG, Degenhardt TP, Schotzinger RJ, McGill JB. Effects of pyridoxamine in combined phase 2 studies of patients with type 1 and type 2 diabetes and overt nephropathy. Am J Nephrol. 2007;27(6):605–14. 17823506 10.1159/000108104

[41] Lewis EJ, Hunsicker LG, Rodby RA. A clinical trial in type 2 diabetic nephropathy. Am J Kidney Dis. 2001;38(4 Suppl 1):S191–S4. 11576953 10.1053/ajkd.2001.27442

[42] Kovacic P, Somanathan R. Cell signaling and receptors in toxicity of advanced glycation end products (AGEs): alpha-dicarbonyls, radicals, oxidative stress and antioxidants. J ReceptSignalTransductRes. 2011;31(5):332–9. 10.3109/10799893.2011.607171 21929288

[43] Blush J, Lei J, Ju W, Silbiger S, Pullman J, Neugarten J. Estradiol reverses renal injury in Alb/TGF-beta1 transgenic mice. Kidney Int. 2004;66(6):2148–54. 15569304 10.1111/j.1523-1755.2004.66005.x

[44] Schiffer M, Bitzer M, Roberts IS, Kopp JB, ten Dijke P, Mundel P, et al Apoptosis in podocytes induced by TGF-beta and Smad7. JClinInvest. 2001;108(6):807–16. 10.1172/JCI12367 PMC200928 11560950

[45] Schnaper HW, Jandeska S, Runyan CE, Hubchak SC, Basu RK, Curley JF, et al TGF-beta signal transduction in chronic kidney disease. Front Biosci. 2009;14:2448–65. 10.2741/3389 PMC4367189 19273211

[46] Poncelet AC, Schnaper HW. Regulation of human mesangial cell collagen expression by transforming growth factor-beta1. AmJPhysiol. 1998;275(3 Pt 2):F458–66. 10.1152/ajprenal.1998.275.3.F458 9729521

[47] Cohen MP, Sharma K, Guo J, Eltayeb BO, Ziyadeh FN. The renal TGF-beta system in the db/db mouse model of diabetic nephropathy. ExpNephrol. 1998;6(3):226–33. 10.1159/000020527 9639038

[48] Goldfarb S, Ziyadeh FN. TGF-beta: a crucial component of the pathogenesis of diabetic nephropathy. TransAmClinClimatolAssoc. 2001;112:27–32. PMC2194397 11413780

[49] Casalena G, Daehn I, Bottinger E. Transforming Growth FactorB, Bioenergetics, and Mitochondria in Renal Disease. Seminars in Nephrology. 2012;32(3):295–303. 10.1016/j.semnephrol.2012.04.009 22835461 PMC3444292

[50] Kume S, Haneda M, Kanasaki K, Sugimoto T, Araki S, Isshiki K, et al SIRT1 inhibits transforming growth factor beta-induced apoptosis in glomerular mesangial cells via Smad7 deacetylation. The Journal of biological chemistry. 2007;282(1):151–8. 10.1074/jbc.M605904200 . 17098745

[51] Minagawa S, Araya J, Numata T, Nojiri S, Hara H, Yumino Y, et al Accelerated epithelial cell senescence in IPF and the inhibitory role of SIRT6 in TGF-beta-induced senescence of human bronchial epithelial cells. American journal of physiology Lung cellular and molecular physiology. 2011;300(3):L391–401. 10.1152/ajplung.00097.2010 21224216 PMC3284316

[52] Yang XJ, Seto E. Lysine Acetylation: Codified Crosstalk with Other Posttranslational Modifications. Molecular Cell. 2008;31(4):449–61. 10.1016/j.molcel.2008.07.002 18722172 PMC2551738

[53] Oh AS, Lorant LA, Holloway JN, Miller DL, Kern FG, El Ashry D. Hyperactivation of MAPK induces loss of ERalpha expression in breast cancer cells. MolEndocrinol. 2001;15(8):1344–59. 10.1210/mend.15.8.0678 11463858

[54] Holloway JN, Murthy S, El-Ashry D. A cytoplasmic substrate of mitogen-activated protein kinase is responsible for estrogen receptor-alpha down-regulation in breast cancer cells: the role of nuclear factor-kappaB. Mol Endocrinol. 2004;18(6):1396–410. 15056731 10.1210/me.2004-0048

[55] Kawahara TL, Michishita E, Adler AS, Damian M, Berber E, Lin M, et al SIRT6 links histone H3 lysine 9 deacetylation to NF-kappaB-dependent gene expression and organismal life span. Cell. 2009;136(1):62–74. 10.1016/j.cell.2008.10.052 19135889 PMC2757125

[56] Yeung F, Hoberg JE, Ramsey CS, Keller MD, Jones DR, Frye RA, et al Modulation of NF-kappaB-dependent transcription and cell survival by the SIRT1 deacetylase. EMBO J. 2004;23(12):2369–80. 15152190 10.1038/sj.emboj.7600244 PMC423286

[57] Lu C, He JC, Cai W, Liu H, Zhu L, Vlassara H. Advanced glycation endproduct (AGE) receptor 1 is a negative regulator of the inflammatory response to AGE in mesangial cells. Proceedings of the National Academy of Sciences of the United States of America. 2004;101(32):11767–72. 15289604 10.1073/pnas.0401588101 PMC511050

[58] Guarente L. Sirtuins, Aging, and Medicine. New England Journal of Medicine. 2011;364(23):2235–44. 10.1056/NEJMra1100831 21651395

[59] Turgut F, Bolton WK. Potential new therapeutic agents for diabetic kidney disease. American journal of kidney diseases: the official journal of the National Kidney Foundation. 2010;55(5):928–40. 10.1053/j.ajkd.2009.11.021 . 20138415

[60] Yu M. Gender differences in chronic kidney disease and progression in type 2 diabetes. Journal of American Society of Nephrology. 2011.

[61] Uribarri J, Cai W, Pyzik R, Goodman S, Chen X, Zhu L, et al Suppression of native defense mechanisms, SIRT1 and PPARγ, by dietary glycoxidants precedes disease in adult humans; relevance to lifestyle-engendered chronic diseases. Amino Acids. 2013:1–9. 10.1007/s00726-013-1502-4 PMC3795943 23636469

